# An Antiviral Drug Screening Platform with a FRET Biosensor for Measurement of Arenavirus Z Assembly

**DOI:** 10.1247/csf.20030

**Published:** 2020-11-13

**Authors:** Tatsuaki Mizutani, Yusuke Ohba, Satoshi Mizuta, Jiro Yasuda, Shuzo Urata

**Affiliations:** 1 Laboratory of Cell Regulation, Institute for Frontier Life and Medical Sciences Kyoto University, 53 Kawahara-cho, Shogoin, Sakyo-ku, Kyoto 606-8507, Japan; 2 Laboratory of Cell Regulation and Molecular Network, Graduate School of Biostudies, Kyoto University, Yoshida-Konoe-cho, Sakyo-ku, Kyoto 606-8501, Japan; 3 Department of Cell Physiology, Faculty of Medicine and Graduate School of Medicine, Hokkaido University, N15W7, Kita-ku, Sapporo 060-8638, Japan; 4 Center for Bioinformatics and Molecular Medicine, Graduate School of Biomedical Sciences, Nagasaki University, 1-12-4 Sakamoto, Nagasaki 852-8523, Japan; 5 Department of Emerging Infectious Diseases, Institute of Tropical Medicine (NEKKEN), Nagasaki University, 1-12-4 Sakamoto, Nagasaki, 852-8523, Japan; 6 National Research Center for the Control and Prevention of Infectious Diseases (CCPID), Nagasaki University, 1-12-4 Sakamoto, Nagasaki, 852-8523, Japan

**Keywords:** Arenavirus, Förster resonance energy transfer, anti-viral drugs, Z protein

## Abstract

The smallest arenavirus gene product, Z protein, plays critical roles in the virus life cycle. Z is the major driving force of budding and particle production because of a unique property that defines self-assembly. In addition to the roles in budding, Z also participates in the suppression of type I interferon production to evade host antiviral immunity. Therefore, Z and its assembled form are an attractive drug target for arenaviral hemorrhagic fever, such as Lassa fever. Here, we developed a biosensor that enabled the evaluation of the prototype arenavirus, lymphocytic choriomeningitis virus (LCMV), Z assembly using the principle of Förster resonance energy transfer (FRET). This FRET biosensor consisted of three tandem Z that were sandwiched between super-enhanced cyan-emitting fluorescent protein and variant of a yellow-emitting mutant of green fluorescent protein so that Z-Z intermolecular binding via the really interesting new gene finger domain increased the emission ratio. To identify novel anti-arenavirus compounds, the FRET biosensor was employed to screen the PathogenBox400 for inhibitors of Z assembly in a 96-well plate format. The assay performed well, with a Z’-factor of 0.89, and identified two compounds that decreased the emission ratio of the FRET biosensor in a dose-dependent manner. Of them, the compound, 5,6,7,8-tetrahydro-7-(benzyl)-pyrido[4',3':4,5]thieno[2,3-d]pyrimidin-2,4-diamine, was found to significantly inhibit LCMV propagation in infected cells. Thereby, the present study demonstrated that a novel FRET biosensor incorporating Z assembly built on FRET and named Zabton, was a valuable screening tool to identify anti-arenavirus compounds in the context of inhibition of Z assembly.

## Introduction

Several arenaviruses are responsible for severe hemorrhagic fever (HF) in humans, such as Lassa fever (LF) and the South American HF (Buchmeier *et al.*, 2013). Although the off-label nucleoside analog drug ribavirin (1-β-D-ribofuranosyl-1,2,4-triazole-3-carboxamide; Rib), is used for the treatment of LF, the mechanism of action of Rib against the viruses is currently not entirely understood (Parker, 2005). In addition, the anti-arenaviral effect of Rib is limited and is associated with a significant side effect (Buchmeier *et al.*, 2013). The lack of FDA-approved drugs represents a severe public health problem, even if these viral HF diseases are endemic in certain countries of West Africa and South America, due to increased travel to and from these endemic regions (Isaacson, 2001).

All arenaviruses encode five mature products: a nucleoprotein (NP), an RNA-dependent RNA polymerase (RdRp or L), the matrix protein (Z), and two envelope glycoproteins (GP1 and GP2) (Buchmeier *et al.*, 2013). Of these viral proteins, the Z protein is composed of three domains, the N-terminal domain, central-domain, and C-terminal domain (Urata and Yasuda, 2012). The glycine at position 2 in the N-terminal domain is conserved among the arenavirus Z proteins and is responsible for the myristoylation required to anchor the host membrane at the budding stage (Perez *et al.*, 2004; Strecker *et al.*, 2006; Urata and Yasuda, 2015). The N-terminal domain is important for antagonizing retinoic acid-inducible gene-I (RIG-I) like receptor-dependent interferon (IFN) production (Xing *et al.*, 2015). The central domain is a really interesting new gene (RING) finger motif, which is a zinc-finger type structural domain and is responsible for assembly (Hastie *et al.*, 2016; Kentsis *et al.*, 2001). This central RING finger domain is essential for the inhibition of viral RNA synthesis (Cornu and de la Torre, 2002; Kranzusch and Whelan, 2011) and the interaction with several host factors (Kentsis *et al.*, 2001). Through a variety of interactions with host molecules, including the promyelocytic leukemia protein (PML) and the eukaryotic translation initiation factor 4E (eIF4E), Z protein regulates host and virus RNA synthesis, as well as protein translation (Kentsis *et al.*, 2001). Importantly, the RING finger domain is highly conserved across all arenavirus species, and contributes to both Z self-assembly and recruitment of cellular proteins (Urata and Yasuda, 2012). The C-terminal of Z protein possesses a late (L)-domain, which is a short amino acid motif critical for budding via interaction with specific host factors (Urata *et al.*, 2006; Urata and Yasuda, 2012; Urata *et al.*, 2009). When arenavirus Z protein is transiently expressed in mammalian cells, Z protein is spontaneously transported to the cell membrane, assembled, attached to the host membrane, and facilitates the formation of virus-like particles (Perez *et al.*, 2003; Strecker *et al.*, 2003; Urata *et al.*, 2006). Naturally, the highly conserved arenaviral Z protein, with many vital functions in viral replication, was envisaged as a promising target for anti-arenavirus infection. Over the past decades, several trials have been reported to acquire novel anti-arenavirus drugs targeting Z protein functions (Garcia *et al.*, 2006, 2010; Lu *et al.*, 2014). To the best of our knowledge, unfortunately, there have been no approved drugs developed that target arenavirus Z protein to date.

Here, we developed a novel FRET-based biosensor to monitor the prototypic arenavirus lymphocytic choriomeningitis virus (LCMV)-Z protein assembly in both living cells and cell lysates. The biosensor encompassed unique properties of Z protein, including zinc-binding, self-assembly, and suitability for high-throughput screening. Preliminary screening with four hundred compounds using the FRET biosensor worked well and acquired potential hit compounds. Through a typical bioassay and an LCMV infection assay, one compound was finally identified, 5,6,7,8-tetrahydro-7-(benzyl)-pyrido[4',3': 4,5]thieno[2,3-d]pyrimidin-2,4-diamine, as a useful candidate for inhibiting arenavirus propagation.

## Materials and Methods

### Cells, compounds and antibodies

Expi293 cells were purchased from Invitrogen and maintained in Expi293 expression medium (Life Technologies, Carlsbad, CA, USA). HeLa and HEK293T cells were obtained from RIKEN and maintained in Dulbecco’s modified Eagle’s medium (DMEM) (Sigma-Aldrich, St. Louis, MO) supplemented with 10% (v/v) fetal bovine serum (FBS, Hyclone, Logan, UT). Vero 76 cells were obtained from the Health Science Research Resources Bank (JCRB9007, Osaka, Japan) and were maintained in DMEM supplemented with 1% penicillin/streptomycin and 10% FBS. The Medicines for Malaria Venture (MMV) Pathogen Box contained 400 compounds as 10 mM stock solutions in dimethyl sulfoxide (DMSO), and was obtained free of charge from MMV (https://www.mmv.org). N,N,N',N'-Tetrakis(2-pyridylmethyl)ethylenediamine (TPEN) was purchased from Dojindo (Kumamoto, Japan). Ethylenediaminetetraacetic acid (EDTA) was purchased from Nacalai Tesque Inc. (Kyoto, Japan). Rabbit antisera against LCMV Z protein was produced by immunizing rabbits with recombinant LCMV Z protein produced in *E. coli* (Eurofins Genomics, Tokyo, Japan), as reported for Lassa virus Z protein (Urata *et al.*, 2006) and Marburg virus VP40 (Urata *et al.*, 2007). An anti-FLAG mouse monoclonal antibody was purchased from Sigma-Aldrich. The mouse immunoglobulin (Ig) G1 isotype control was obtained from BioLegend (San Diego, CA, USA). Anti-green fluorescent protein, anti-β actin rabbit polyclonal antibodies, and the horseradish peroxidase (HRP)-conjugated anti-rabbit IgG secondary antibody were purchased from Cell Signaling Technology (Danvers, MA, USA). Anti-LCMV NP rat-IgG antibody (clone VL-4, BioXCell, West Lebanon, NH, USA) and Alexa Fluor 488-labeled anti rat-IgG antibody (Abcam, Cambridge, MA, USA) were used for LCMV titration.

### Expression vector for the FRET biosensor

**Fig. 1:**
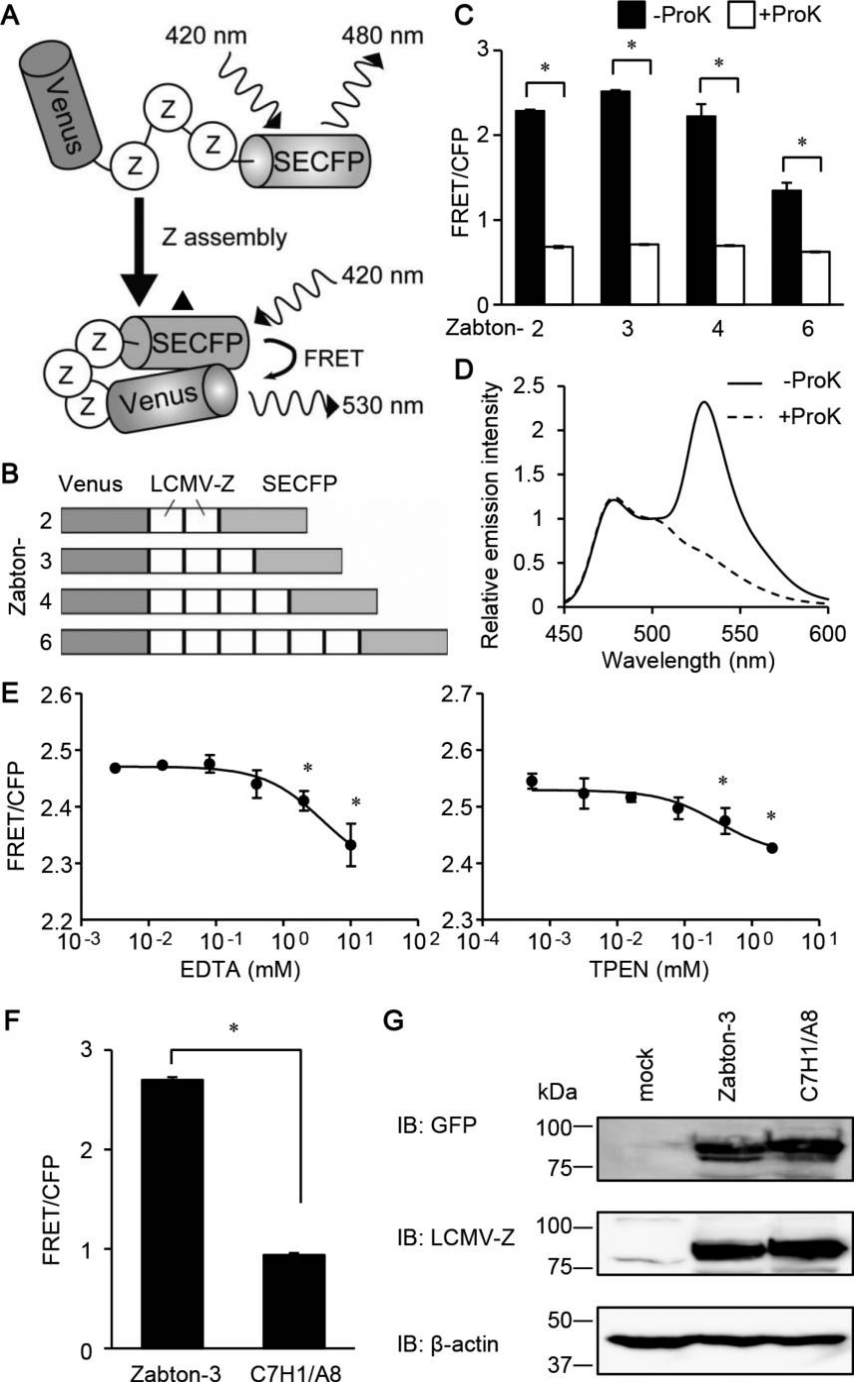
Development of FRET biosensor to monitor Z protein assembly. (A) Schematic representation of a biosensor to monitor the assembly of lymphocytic choriomeningitis virus (LCMV)-Z protein, named as Zabton. Briefly, Zabton consists of a variant of Venus, LCMV-Z protein, and SECFP from the NH_2_ terminus. The Z-Z interaction causes an assembled protein complex, which increases the efficiency of FRET. (B) Domain structures of Zabton-2, 3, 4, and 6. (C) Emission ratios (FRET/CFP) of Zabton-2, 3, 4, and 6 expressed in Expi293 cell lysates, with or without proteinase K treatment. After 24 hours of transfection, the cells were analyzed using fluorescence spectrometry at an excitation wavelength of 420 nm. Fluorescence emission was expressed as the mean emission ratio±SD of pooled data obtained from three separate experiments. (D) Emission spectra of Zabton-3 expressed cell lysates treated with (dashed line) or without (solid line) proteinase K. Relative emission intensity was normalized value by the emission intensity at 500 nm. (E) Emission ratios of Zabton-3 expressed in Expi293 cells with EDTA (left) or TPEN (right) with 0.016–10 mM treatment. *, *p*<0.05; values are determined by ANOVA with Dunnett’s test among mock-treated, and the ion chelator-treated groups. (F) Emission ratios of Zabton-3 and -3^C7H1/A8^ expressed in Expi293 cell lysates. (G) Cell lysates derived from Expi293 cells transfected with either Zabton-3 or -3^C7H1/A8^ were resolved using SDS-PAGE, and immunoblotted with antisera against LCMV Z protein, and antibodies to green fluorescent protein and β-actin.

Expression vectors for Venus, a variant of a yellow-emitting mutant of green fluorescent protein, and SECFP, a super-enhanced cyan-emitting fluorescent protein, were kindly provided by A. Miyawaki (RIKEN). Zabton was generated using the pCAGGS eukaryotic expression vector; it encoded a chimeric protein that consisted of Venus, the 5× GS linker (Gly-Gly-Gly-Gly-Ser), multiple LCMV-Z proteins, the 5× GS linker, and SECFP from the amino terminus (see Fig. 1B). The coding region of Venus was amplified by polymerase chain reaction. The resulting Venus fragment was subcloned into the EcoRI/NotI sites of pCAGGS-SECFP to obtain pCAGGS-Venus-SECFP. For the construction of the multiple Z protein-encoding vectors, the open reading frame of the LCMV-Z protein with N-terminal XhoI and BamHI sites and C-terminal BglII and NotI sites were synthesized and inserted into pIDTSMART-AMP to generate pIDT-Z (IDT Technologies, Coralville, IA, USA). The pIDT-Z vector was digested by BamHI and NotI, cloned into the BglII-NotI site of pIDT-Z to obtain pIDT-Zx2. In a similar manner, pIDT-Zx3, -Zx4, and -Zx6 were generated. The multiple Z protein-encoding plasmids were digested by XhoI and NotI, followed by subcloning of multiple LCMV-Z proteins into XhoI/NotI sites of pCAGGS-Venus-SECFP, to obtain an expression vector for the Z protein assembly indicator. Mutations in the zinc motifs of LCMV-Z protein (referred to as the C7H1/A8 mutant) containing the following mutations: C32A, C35A, C45A, H48A, C51A, C54A, C65A, and C68A were synthesized by IDT Technologies, and cloned into the pIDTSMART vector.

### Transfection

Expi293 cells were transfected with Zabton or Zabton^C7H1/A8^ expression vectors using the ExpiFectamine 293 transfection reagent (Life Technologies), according to the manufacturer’s protocol. At 72 hours post-transfection, cells were harvested by centrifugation, lysed in lysis buffer [20 mmol/L Tris-HCl (pH 7.5), 100 mmol/L NaCl, and 1% Triton-X], clarified by additional centrifugation, and subjected to spectroscopy, as well as immunoblotting. Gene transfer into HeLa cells was performed using Lipofectamine 3000 transfection reagents (Gibco, Carlsbad, CA, USA).

### Fluorescence measurement

The fluorescence of Zabton probes expressing cell lysates were measured using a SpectraMax i3x multimode microplate reader (Molecular Devices, Sunnyvale, CA, USA) or an ARVO-X3 (PerkinElmer, Waltham, MA, USA). The fluorescence emissions of SECFP (480 nm±10 nm) and FRET (530 nm±10 nm) at an excitation wavelength of 420 nm±10 nm were measured with a SpectraMax i3x multimode microplate reader. With an ARVO-X3 system, the fluorescence emissions of SECFP (460 nm) and FRET (535 nm) at an excitation wavelength of 406 nm were measured. After background subtraction, the emission ratio of FRET and SECFP (FRET/CFP) was calculated. A fluorescence spectrum was obtained by means of an FP-6500 fluorescence spectrometer (JASCO Co.), with an excitation wavelength of 420 nm. For the demonstration of FRET, the cell lysates were incubated with 50 μg/ml proteinase K (Promega, Madison, WI, USA) at 37°C for 30 min and analyzed with the fluorescence microplate reader, or the spectrometer. Fluorescence measurement of the probes was performed with a SpectraMax i3x multimode microplate reader unless otherwise indicated.

### Screening of the Pathogen Box compounds

The 400 compounds of the Pathogen Box were prepared according to the MMV foundation instructions, and screened on 96-well plates (Corning, Acton, MA, USA) using Zabton-expressing Expi293 cell lysates. The lysates (50 μL) were arrayed into 96-well plates, and 50 μL of the working stock (20 μM, diluted from the provided stock (10 mM) with DMSO) was added to each well of the 96-well plate, resulting in a final screening concentration of 10 μM. DMSO (0.05%) was used as a negative control. The fluorescence emissions of SECFP and FRET were obtained by means of a SpectraMax i3x multimode microplate reader. The quality of the screen was evaluated using the Z’-factor, signal to background (S/B) and signal to noise (S/N) based on the difference in the emission ratios between Zabton^C7H1/A8^ and Zabton-expressing samples as negative (0%) and positive (100%) controls, respectively. Z’=1–{(3·Standard deviation (S.D.)_100%_+3·S.D._0%_)/(Mean_100%_–Mean_0%_)}. S/B=Mean_100%_/Mean_0%_. S/N=(Mean_100%_–Mean_0%_)/S.D._0%_

### FRET imaging

HeLa cells expressing Zabton were cultured in phenol red-free DMEM (Invitrogen) buffered with 15 mM 4-(2-hydroxyethyl)-1-piperazineethanesulfonic acid) (HEPES, pH 7.4 to avoid CO_2_ control) and plated on a collagen-coated glass base plate (Asahi Techno Glass, Tokyo, Japan). Cell image acquisition was performed, as previously described (Mizutani *et al.*, 2010). Briefly, the cells were imaged with an IX81 inverted microscope (Olympus, Tokyo, Japan) equipped with an automated XY-stage (Chuo Precision Industrial, Tokyo, Japan), a stage-top incubation chamber at 37°C (Live Cell Instrument, Seoul, Korea), an MAC6000 filter and a shutter control unit (Ludl Electronic Products, Hawthorne, NY, USA), and a Cool SNAP MYO cooled charge-coupled device camera (Photometrics, Tucson, AZ, USA) controlled with the MetaMorph software (Universal Imaging, West Chester, PA, USA). The following filters were obtained from Semrock (Rochester, NY, USA): FF02-438/24-25 excitation and FF01-483/32-25 emission filters for SECFP, FF01-504/12-25 excitation and FF01-542/27-25 emission filters for Venus, and FF02-438/24-25 excitation and FF01-542/27-25 emission filters for FRET. An FF458-Di02-25x36 dichroic mirror (Semrock) was used throughout the experiments. The cells were illuminated with a SOLA Light Engine (Lumencor, Beaverton, OR, USA) and imaged through a 60× immersion objective lens (numerical aperture, 1.35). The exposure times for 4×4 binning were 200 ms for SECFP, Venus, and FRET images and 50 ms for differential interference contrast images. After background subtraction, ratio images of FRET and SECFP were created using the MetaMorph software (Universal Imaging, West Chester, PA, USA) and were represented by the intensity-modulated display (IMD) mode. In the IMD mode, eight colors from red to blue were used to represent the emission ratio of FRET and SECFP (FRET/CFP), with the intensity of each color indicating the mean intensity of FRET and SECFP. For quantification, the FRET and SECFP intensities were averaged over the whole cell area, exported to Excel software (Microsoft Corporation, Redmond, WA, USA), and used to calculate the emission ratio.

### Immunoblotting

The cell lysates were diluted in 6× Laemmli sample buffer (Nacalai Tesque Inc.), incubated at 95°C for 5 min, and then, the samples were subjected to sodium dodecyl sulfate (SDS)-polyacrylamide gel electrophoresis (PAGE). The separated proteins were transferred to a polyvinylidene difluoride membrane (Millipore, Darmstadt, Germany) and subjected to immunoblot analysis using antibodies indicated in the figure legends. Proteins were detected using the Enhanced Chemiluminscence (ECL) Western Blotting Detection Reagent (GE Healthcare, Little Chalfont, UK) and a LAS-3000 UV mini image analyzer (Fujifilm, Tokyo, Japan), as previously described (Yoshioka *et al.*, 2016).

### Viral titration

An immunofocus assay was performed to determine LCMV titers. Vero 76 cells (3×10^4^ cells) were seeded into a 96-well plate one day before infection. The cells were infected with 1:10 serial dilutions of the viruses, and incubation was continued for 16 hours at 37°C and 5% CO_2_. The cells were then fixed with 4% paraformaldehyde (PFA) for 30 min at room temperature and incubated with phosphate-buffered saline (PBS) containing 0.1% Tween 20 for 1 hour at room temperature. Blocking with 10% FBS in dilution buffer (3% bovine serum albumin and 0.3% Triton X-100 in PBS) was performed at 4°C overnight. Fixed cells were stained with anti-LCMV NP antibody and fluorescein isothiocyanate-conjugated anti-rat IgG antibody for 3 hours each at room temperature. The LCMV NP-positive cells were visualized using an AxioVert A1 microscope (Carl Zeiss Meditec, Jena, Germany), and used to determine the fluorescent focus unit/mL, as the virus titer.

### Evaluation of the selected compounds on LCMV propagation

The Armstrong strain of recombinant LCMV (rLCMV) was rescued using the reverse genetics method and propagated in Vero 76 cells (Flatz *et al.*, 2006). HEK293T cells were infected with rLCMV at 0.5 multiplicity of infection (MOI). At 1.5 hours post-infection, the viral inoculum was removed and fresh media containing different concentrations of compounds, or vehicle, were added. At 24 hours post-infection, culture supernatant was collected to measure viral titration, as described above.

### Cytotoxicity assay

The effect of hit compounds on cell viability was determined using the CellTiter-Glo Luminescent Cell Viability assay (Promega, Madison, WI, USA). Briefly, HEK293T cells were seeded into 96-well plates (2×10^4^ cells/well) and treated with increasing concentrations of compounds (0, 20, or 50 μM). At 24 h post-treatment, cells were incubated with the CellTiter-Glo reagent, and the assay was performed according to the manufacturer’s recommendations.

### Quantification and Statistical Analysis

Excel and GraphPad Prism 5 (GraphPad Software, Inc., San Diego, CA. USA) software were used for all statistical analyses. Quantitative data are presented as the mean ±S.D. from at least three independent experiments (unless otherwise indicated). For all calculations, *p*<0.05 was considered significant and was represented using an asterisk. Group comparisons were performed using one-way analysis of variance (ANOVA), followed by Dunnett’s multiple comparisons test. Welch’s *t*-test was used as a comparison between two groups.

## Results

### Development of a FRET-based biosensor to monitor Z protein assembly

Previous reports have demonstrated that LCMV Z proteins are self-assembled in mammalian cells after transfection with LCMV-Z protein-encoding plasmid DNA alone (Kentsis *et al.*, 2002; Urata *et al.*, 2006). Accordingly, we designed a protein in which several Z proteins were sandwiched between Venus and SECFP to develop a new FRET-based biosensor for monitoring Z protein assembly. This biosensor was expected to increase the FRET efficiency upon Z-Z intermolecular assembly, and was named Zabton: Z assembly based on FRET for screening (Fig. 1A and B). To confirm that the increased emission ratio was caused by FRET, a Zabton series was digested with proteinase K. Since green fluorescence protein mutants, Venus and SECFP, are exceptionally resistant to proteinase K, this treatment will generate free forms of Venus and SECFP (Miyawaki and Tsien, 2000). After treatment with proteinase K, the fluorescence intensity at 530 nm excited at 475 nm remarkably decreased in all Zabton series, demonstrating that these signals were caused by FRET (Fig. 1C). Among the four biosensors constructed using multiple Z proteins, Zabton-3 (trimeric tandem Z protein) exhibited the highest FRET/CFP emission ratio (Fig. 1C and D). We also noticed that some numeric Zabtons exhibited higher S.D. than Zabton-3, which was most likely due to the aggregation. Totally, Zabton-3 was superior to the other Zabton series in S/N ratio and statistical variability. As shown previously, RING finger domains of the LCMV-Z protein is required for the assembly of the protein (Kentsis *et al.*, 2002). Indeed, destabilization of the zinc-binding sites in the RING finger domain of LCMV-Z protein influences the stability and folding of Z protein, resulting to decrease in the capacity of self-assembly (Kentsis *et al.*, 2001, 2002). Thus, we next examined if the emission ratio of Zabton-3 depended on the RING finger domain. First, we treated Zabton-3 with the metal ion chelators, EDTA or TPEN, to disrupt the ion coordination in the RING finger domain. We found that the emission ratio of Zabton was modestly reduced in the presence of either chelator, in a dose-dependent manner (Fig. 1E). A partial inhibition of the RING finger domain did not significantly affect the Z assembly, as previously reported (Wang *et al.*, 2012). To further examine the effect of the RING finger domains on FRET, we created a mutant of Zabton-3 harboring an alanine substitution at the central Cys3HisCys4 motif in the RING finger domain, referred to as Zabton-3^C7H1/A8^. The emission ratio of Zabton-3^C7H1/A8^ was significantly reduced compared to that of the wild-type (WT) Zabton-3 (Fig. 1F). Immunoblotting experiments revealed that the expression levels of Zabton-3 and Zabton-3^C7H1/A8^ were comparable in the experimental conditions (Fig. 1G). These observations highlighted that the assembly of Z protein via the RING finger domain was critically important for the FRET observed with Zabton-3.

### Screening of compounds using the Zabton-based FRET assay

**Fig. 2:**
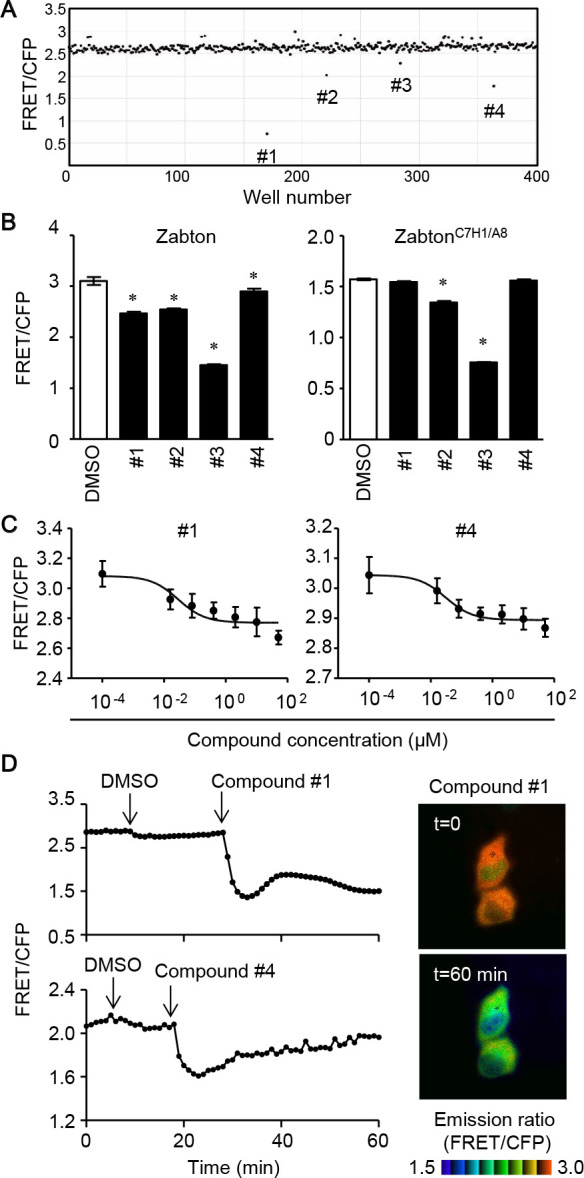
Compound screening with Zabton identifies hit compounds. Four hundred compounds from Pathogen Box were screened to identify inhibitory compounds of Z protein self-assembly using Zabton in 96-well plates, as described in the Materials and Methods. (A) The dot plot of the FRET/CFP ratio of Zabton with Pathogen Box 400 compounds at 10 μM. (B) Emission ratios of Zabton or Zabton^C7H1/A8^ expressed in Expi293 cells with different compounds at 10 μM treatment analyzed by ARVO x3 fluorescence spectrometry. *, *p*<0.05; values were determined by ANOVA with Dunnett’s test among mock-treated and compound-treated samples. (C) Zabton-expressing cell lysates, treated with two hit compounds (0.0001–50 μM) for 30 min as indicated, and analyzed by ARVO x3 system. Values were expressed as a relative emission ratio in arbitrary units for the mean±SD. (D) Responses of HeLa cells expressing Zabton upon the addition of DMSO, followed by the addition of 5 μM compounds. All traces represent the average of at least four cells of the emission ratio (left). Right, the emission ratio and the intensity of SECFP was used to generate reconstituted images in IMD mode, and photographs before (top, t=0) and 60 min after compound #1 treatment (bottom, t=60) were shown. Representative results for at least two separate experiments are shown.

To examine if Zabton-3 was suitable for identifying compounds that inhibit Z protein assembly, we calculated the Z’ factor between Zabton-3 and Zabton-3^C7H1/A8^, which was 0.89, with a signal-to-background of 3.21, and a signal-to-noise of 246.1 in a 96-well format (Fig. 1E, and Materials and Methods). Accordingly, we determined that Zabton-3 (hereafter, referred to as Zabton) was a valuable tool to monitor LCMV Z protein assembly via RING finger domains in a high-throughput screening assay. Using the Zabton-based FRET assay, we screened the Pathogen Box library of 400 small molecules. The library contained drug-like molecules active against neglected diseases and structurally diverse compounds, including a set of currently marketed drugs (for details, see Materials and Methods). Compounds were screened at a final concentration of 10 μM in a volume of 100 μL Zabton-expressing cell lysate per well (Fig. 2A). Hits (#1–#4) were defined as compounds that decreased the emission ratio of Zabton by greater than three times the standard deviation (SD) for compound responses in each plate, with reproducibility (Fig. 2B). Although four hits were identified from the first screen, we noticed that two compounds (#2 and #3) significantly decreased the FRET ratio of the assembly deficient Zabton^C7H1/A8^. Notably, the inhibitory efficacies on Zabton^C7H1/A8^ with compound #3 (51.9±0.2%) were comparable to that of WT-Zabton (53.1±0.4%) (Fig. 2B). Additionally, we noticed that compound #2 had significant auto-fluorescence at 414 nm, and that #3 exhibited a strong emission at 480 nm with 405 nm excitation (Supplementary Tables 1 and 2). Unlike these false-positive compounds (#2 and #3), compounds #1 and #4 did not exhibit obvious auto-fluorescence detected in the CFP and FRET channels (Supplementary Tables 1 and 2). With regard to the remaining two compounds, #1 and #4, we found both of them decreased the FRET ratio in a concentration-dependent manner and compound #1 exhibited higher inhibition magnitudes than compound #4 in terms of the FRET/CFP emission ratio. Interestingly, these hit compounds are known to have different antibacterial activities as follows, MMV688547 (#1) has demonstrated activity against *Trypanosoma brucei* (Duffy *et al.*, 2017) and MMV688345 (#4) has an inhibitory effect on *Plasmodium falciparum* (Rosowsky *et al.*, 1973; Valenciano *et al.*, 2019). Based on the results of FRET inhibition using cell lysates, we next monitored the effect of supplementation with each hit compound on live cells. As shown in Fig. 2D, live imaging with time-lapse dual-emission fluorescence microscopy highlighted that the extracellular addition of the compound continuously decreased the FRET/CFP ratio of Zabton in HeLa cells. Therefore, Zabton seemed to be a valuable tool for alternative screening of anti-arenavirus drugs through live-cell imaging.

### Hit compounds inhibited LCMV replication.

**Fig. 3:**
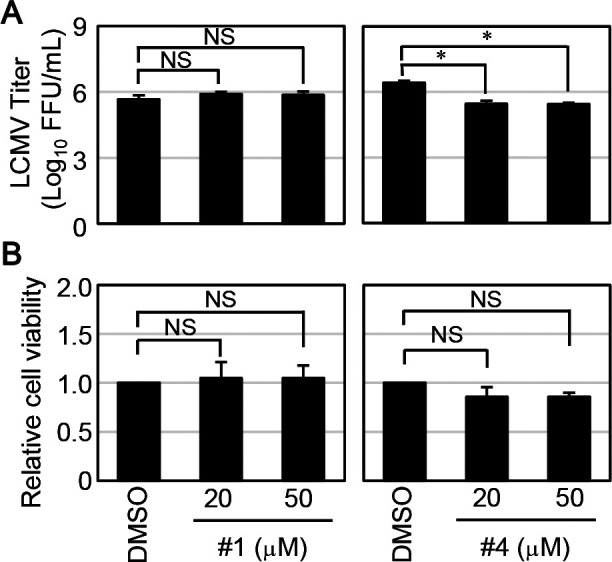
Hit compounds inhibit LCMV replication and propagation. (A) HEK293T cells were infected with LCMV at a multiplicity of infection (MOI) of 0.5. At 1.5 hours post-infection (p.i.), the culture media were replaced with fresh media containing DMSO, 20, or 50 μM of either compound #1 or #4. Viral titers in culture media collected at 24 hours p.i. were determined. The data were averages and SD from three independent experiments. (B) HEK293T cells were treated with the indicated concentrations of either compound #1 or #4. At 24 hours post-treatment, cell viability was measured, as described in the Materials and Methods. Dunnett’s method was used to compare the difference statistically between the means of compounds’ treated groups and the mean of a control DMSO treatment group as described in the Materials and Methods (NS; not significant, *; *p*<0.05).

Next, we evaluated the effect of the hit compounds from the FRET-based screening assay on LCMV propagation. While the treatment of compound #1 on LCMV-infected cells did not show any inhibitory effect of virus propagation, the production of infectious viruses from compound #4 treated LCMV-infected cells (both 20 and 50 μM) was reduced compared to that of DMSO-treated LCMV-infected cells (Fig. 3A). Since the reduction of LCMV production by the treatment of compound #4 was statistically significant, we also verified the compound’s cell toxicity. As shown in Fig 3B, the treatment of compound #4 exhibited a slight decrease in cell viability without statistical significance. Thus, we concluded that #4 had an inhibitory effect on LCMV propagation.

## Discussion

Novel screening of 10 μM compounds using a FRET biosensor named Zabton identified two hits after a cut-off of three SDs from the average of the negative control and the exclusion of compounds through a validation assay. Out of the two selected compounds, one compound #4, (5,6,7,8-tetrahydro-7-(benzyl)-pyrido[4',3':4,5]thieno[2,3-d]pyrimidin-2,4-diamine) exhibited inhibitory activity against LCMV propagation at 20 μM. In the present study, we thus demonstrated that the screening method using the novel FRET sensor Zabton was a promising platform for the identification of effective drugs against arenavirus replication and propagation.

Since the arenaviral Z protein is not only a structural component of the virus, but also interacts with cellular proteins to disorder the host immune system, the Z protein is an attractive drug target for arenavirus infectious diseases (Urata and Yasuda, 2012). Of note, several active compounds have demonstrated the involvement of the reactive compound with the RING finger domain, which is a highly conserved domain among arenaviruses (Garcia *et al.*, 2006, 2010). Although some compounds exhibited evidence for a remarkable effect on virus replication, the mechanism of action in those compounds frequently affected the zinc-coordination of host cellular proteins, which were essential for host homeostasis. Our present study suggested that compound #4 was most likely not affecting zinc coordination with the Z protein, because the emission ratio from other Zabton types, in which FRET was most likely dependent on zinc-binding, was not decreased in the presence of compound #4 (data not shown). Compound #4, thereby, had a unique mechanism of action that might disrupt the precise structure of the Z protein-trimer, which would have much less side effects on the host than previous zinc-finger targeting prodrugs. Arenavirus Z protein has been shown to interact with several host protein partners, including eIF4E and PML (Kentsis *et al.*, 2001). Through the interaction with eIF4E, Z protein selectively represses protein expression involved in host innate immunity, such as IFN regulatory factor 7, which is a master regulator of type-I IFNs (Colina *et al.*, 2008; Honda *et al.*, 2005). As the assembled formation of the Z protein was critical for the interaction with these host proteins, the trimeric conformation might form a favorable motif to associate with the individual host protein. Identification of the partners that specifically interact with trimeric Z protein may reveal the mechanism of action of compound #4, which significantly inhibited LCMV production.

Zabton-based screening identified two-hit compounds (#1 and #4), but the virus propagation was only inhibited by compound #4, not by #1. Although further experiment will be required to clarify the different outcome, there are several possibilities which might explain this controversial result. Arenaviral Z protein has been known to interact with the other viral components, including NP, L, and GPC, during the virus infection (Capul *et al.*, 2007; Loureiro *et al.*, 2011; Ortiz-Riano *et al.*, 2011). Such complicated structures may establish the steric barriers that block the compound’s recruitment into the inhibitory site of Z. Another possibility was raised from the spatial regulation of the Z assembly during the virus infection. Junin virus, one of the arenavirus species, replicates in the characteristic membrane structures called the Replication-Transcription complex (RTC) (Baird *et al.*, 2012). Since the RTC detected in puncta in the cytoplasm, the compound should correctly reach to where Z assemble.

The elegant crystal structure of the Lassa virus Z protein provides evidence that the dodecameric form is built based on dimer blocks (Hastie *et al.*, 2016). Hence, disruption of the Z protein dimer structure would be a more convincing drug target with profound anti-assembly activity, resulting in the suppression of virus production. Indeed, we developed several Zabton series other than Zabton-3. Zabton -2, -4, and -6 all increased the emission ratio, and these are most likely dependent on the dimerized self-assembly property of Z protein. Interestingly, we found that the emission ratio of these Zabtons was not influenced by compound #4 treatment (data not shown). These “even-numeric” Zabton probes are expected to facilitate the identification of other useful compounds with an inhibitory effect on Z protein dimer formation. The detailed binding mode between the compound #4 and the Z-oligomer is not fully explored and should be revealed in the future studies.

As the present study was performed using a preliminary small-scale screen, we found only one hit compound with a pyrimidine ring (Table I) that is found in a variety of natural products, including nucleotides, vitamin B1 and drugs (Li, 2013). Many pyrimidine-related compounds exhibit striking biological activities, including anti-malarial, anti-cancer, and anti-HIV activities (Ajmal *et al.*, 2014; Li, 2013). By proceeding with a larger scale high-throughput screening, i.e., 384-well platforms with >10,000 compounds, it is feasible to acquire useful compounds targeting Z protein assembly and also inhibit LCMV propagation. The principle we revealed here could be further applied to identify novel antiviral compounds against other highly pathogenic arenaviruses, such as Lassa virus, Junin virus, and Lujo virus.

## Author contribution

Conceptualization, T.M. and S.U.; investigation, T.M., Y.O., S.M., J.Y., and S.U.; writing, T.M. and S.U.; funding acquisition, T.M. and S.U.; supervision, T.M. and S.U.

## Conflict of interest

The authors declare that there are no conflicts of interest.

## Figures and Tables

**Table I:** Hit compound, #4, with inhibitory activity of FRET/CFP ratio from the Pathogen Box 400 library.

Compound ID	Chemical formula (Molecular weight)	Structure
#4	C16H17N5S (311.4)	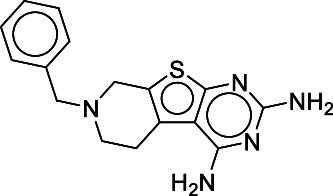

Chemical formula and structure of compound #4 (MMV688345), 5, 6, 7, 8-tetrahydro-7- (benzyl) -pyrido [4', 3':4, 5] thieno [2, 3-D] pyrimidin-2, 4-diamine, is shown.

## Abbreviations

DAPI: 4',6-diamidino-2-phenylindole
DMSO: dimethyl sulfoxide
FBS: fetal bovine serum
FRET: förster resonance energy transfer
GP: glycoprotein
HF: hemorrhagic fever
HRP: horseradish peroxidase
IFN: interferon
Ig: immunoglobulin
LCMV: lymphocytic choriomeningitis virus
LF: Lassa fever
NP: nucleoprotein
PAGE: polyacrylamide gel electrophoresis
PBS: phosphate-buffered saline
PFA: paraformaldehyde
Rib: ribavirin (1-β-D-ribofuranosyl-1,2,4-triazole-3-carboxamide)
RING: really interesting new gene
RdRp: RNA-dependent RNA polymerase
SECFP: super-enhanced cyan-emitting fluorescent protein
TPEN: N,N,N',N'-Tetrakis(2-pyridylmethyl)ethylenediamine
Venus: variant of a yellow-emitting mutant of green fluorescent protein
Zabton: Z assembly built on FRET
