# Supplementary material for: An Antiviral Drug Screening Platform with a FRET Biosensor for Measurement of Arenavirus Z Assembly
Source: Cell Struct Funct. 2020 Nov 13;45(2):155–63. doi: 10.1247/csf.20030 (PMC10511043; doi:10.1247/csf.20030)
Supplement: Supplementary file 1 — Table S1 [file csf_45_20030_1.pdf]

Supplementary materials

Table S1. Absorptions (λAbs) of compounds #1-4. Absorption (λAbs) was measured using a JASC V670DS UV visible near-infrared spectrophotometer. The compounds #1-4, as indicated below, were dissolved in DMSO to make a stock solution at 20 mM. UV–visible and fluorescence spectra were measured using a Shimadzu RF-1500 spectrophotometer. The spectroscopic experiments were performed in a solution (DMSO). The fluorescence emission spectra of #2 were recorded at 414 nm with 370 nm as the excitation wavelength (steady excitation).

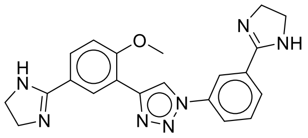

#1

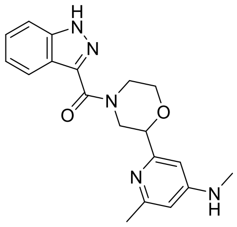

#2

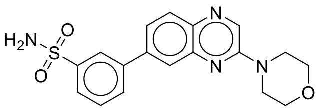

#3

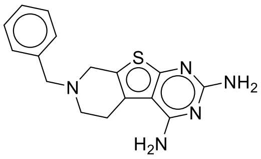

#4

compound	λAbs (nm)	λem(nm)
#1	258	—
#2	282; 370	414
#3	281; 386.5	—
#4	258; 294	—
