# Supplementary material for: An Antiviral Drug Screening Platform with a FRET Biosensor for Measurement of Arenavirus Z Assembly
Source: Cell Struct Funct. 2020 Nov 13;45(2):155–63. doi: 10.1247/csf.20030 (PMC10511043; doi:10.1247/csf.20030)
Supplement: Supplementary file 2 — Table S2 [file csf_45_20030_2.pdf]

Table S2. Fluorescence properties of the compounds #1-#4.

The ten μM of compounds' fluorescence intensity was recorded at 460 and 535 nm with 405 nm as the excitation wavelength using a fluorescence multiplate reader. The compound #2 and #3 detected significant emission at both 460 and 535 nm.

]

compound	Em: 460 nm	Em: 535 nm
None (DMSO)	1035	495
#1	1045	509
#2	8120	6584
#3	26882	7605
#4	1077	535
